# Seasonality of Suicides among Victims Who Are under the Influence of Alcohol

**DOI:** 10.3390/ijerph16152806

**Published:** 2019-08-06

**Authors:** Dorota Lasota, Witold Pawłowski, Paweł Krajewski, Anna Staniszewska, Krzysztof Goniewicz, Mariusz Goniewicz

**Affiliations:** 1 Department of Experimental and Clinical Pharmacology, Medical University of Warsaw, Banacha 1b Street, 02097 Warsaw, Poland; 2 Department of Disaster Medicine, Medical University of Warsaw, Żwirki i Wigury 61 Street, 02091 Warsaw, Poland; 3 Department of Forensic Medicine, Medical University of Warsaw, Oczki 1 Street, 00001 Warsaw, Poland; 4 Department of Security Studies, Polish Air Force Academy, Dywizjonu 303 35 Street, 08521 Dęblin, Poland; 5 Department of Emergency Medicine, Medical University of Lublin, Staszica 4-6 Street, 20081 Lublin, Poland

**Keywords:** suicide, seasonality, alcohol dependence

## Abstract

Introduction: Suicide is one of the most frequent causes of death. According to the World Health Organization (WHO), each year, over eight hundred thousand people worldwide die as a result of suicide. The most common risk factors for suicide are depressive disorders and alcohol dependence. Alcohol can directly influence a decision about suicide, or be a factor facilitating this decision. The aim of the study was to analyse the seasonality of suicides among persons under the influence of alcohol. Material and Methods: Data for analysis were obtained from the Department of Forensic Medicine (DFM) of the Medical University of Warsaw. A retrospective analysis was performed on 317 victims of suicides by hanging, those which were entered into the registry of deaths kept by the DFM in the years 2009–2013. The analysis took into account the age and sex of victims, initial cause of death, date of post-mortem examination, autopsy result and alcohol concentration in the blood or muscles of the victims. Statistical analysis was performed using IBM SPSS Statistics version 20. Results: In the analysis, a spring peak of suicides was found for men, and an autumn peak was revealed for women. In addition, a significant correlation was observed between the age of victims and the concentration of alcohol; the older the victims, the higher the alcohol concentration. However, this correlation was reported only in the spring months. Conclusions: The results of the analysis seem to be consistent with seasonal patterns observed in other studies, and they indicate the occurrence of suicide seasonality. In order to improve the strategies of suicide prevention, it is necessary to identify factors which are related to the seasonal variation of suicidal behaviours, as well as to gain knowledge about the mechanisms behind this phenomenon.

## 1. Introduction

The World Health Organization (WHO) estimates that in 2020, over 1 million people will die as a result of suicide. In Poland, each day, fifteen people make a suicide attempt, and eleven people die by suicide [1]. According to the data of the Polish Central Police Headquarters, more people die from suicide than die as a result of transport accidents. In 2016, there were 9861 suicide attempts, of which 5405 people took their own lives. While at the same time, over 3000 people died in road accidents [2]. In Poland, the most common way to die by suicide is hanging, mainly among men. However, intoxication was more common in women than in men. Women are more likely to attempt suicide, or to engage in non-fatal suicidal behaviour, while men more often take their lives [3].

Suicide is the result of a complex interaction of risk factors and the presence of triggering factors, among which sudden mental disorder, alcohol intoxication and other psychoactive substances, life events, deterioration of somatic and family and personal crisis, and external environmental factors, e.g., weather, are mentioned.

The nervous system is exposed to “psychological” reactions in response to the weather. Meteotropic weather situations include, among others, low-pressure systems and associated sudden changes in particular meteorological elements (atmospheric pressure and temperature) during the passage of atmospheric fronts, especially a cold front, as well as the occurrence of mountain wind and storms. The people most sensitive to weather changes are people suffering from depression and schizophrenia, as well as neurotics, addicts and alcoholics. Among these people, the weather may influence their decision to commit suicide, although it is certainly not the only and decisive reason for taking such a step [4,5,6].

The risk of suicide is estimated at 4% in patients with mood disorders, 7–18% in patients with alcohol dependence [7,8,9], 8% in patients with bipolar affective disorder, and 5% in patients with schizophrenia. Suicide under the influence of alcohol and other psychoactive substances accounts for 25–50% of all suicide cases—the risk of suicide increasing with comorbidities of psychiatric disorders. The risk of suicide in patients with alcohol dependence is 60–120 times greater than in the general population [10].

The aim of the study was to analyse the seasonality of suicides among persons under the influence of alcohol. The study was also based on the results of research by the Central Statistical Office conducted in the years 1999–2003, which confirmed the occurrence of the seasonality of suicides in Poland.

## 2. Material and Methods

Data for analysis were obtained from the Department of Forensic Medicine (DFM), of the Medical University of Warsaw. A retrospective analysis was performed on 317 (75.12%) out of 422 suicide cases entered into the registry of deaths kept by the DFM in the years 2009–2013. The remaining forty victims were rejected due to post-mortem presence of endogenous alcohol, and sixty-five due to missing information. The following parameters were considered in the data analysis: sex, age of victims, date of post-mortem examination, the cause of death confirmed in post-mortem examination, and the level of ethanol in the blood and muscles of victims (BACs (blood alcohol concentration) greater than zero were considered positive) confirmed in a toxicology test, performed with gas chromatography.

In order to answer the research questions, statistical analyses were conducted using the IBM SPSS Statistics version 20 (IBM Corp., Armonk, NY, USA). An analysis of the basic descriptive statistics together with the Kolmogorow–Smirnov test, examining the normality of the distribution, frequency analysis, correlation analysis, Student’s t trial tests, were performed. Independent, one-factor analysis of variance with a posteriori comparisons and chi square independence tests were also calculated. The strength of the contrast analysis effect was calculated from the following formula for Cohen’s d-strength ratio—d = 2t/√df.

## 3. Results

### 3.1. Characteristics of the Studied Groups

A relatively even distribution of reported suicide deaths was observed in the subsequent months and years covered by the study. Of the total cases of suicide, 91.2% were men. Over 96% reported suicides were hangings, confirmed by the presence of a ligature mark in the dissection study (Table 1). The mean age in years was 41.97 ± 13.52. The mean alcohol concentration was 1.71% ± 0.92% and ranged from 0.2–4.4%.

### 3.2. Gender and the Concentration of Ethyl Alcohol

Student’s *t*-test for independent samples did not reveal any significant differences between genders in the range of alcohol concentration—t (315) = 0.719, *p* = 0.473, d = 0.142. The average alcohol concentration in women was not much higher (M = 1.83; SD = 1.03) than the average alcohol concentration in men (M = 1.69; SD = 0.91). Due to the strong counter to the assumption about the parity of groups, the obtained results were confirmed by the Mann–Whitney test. The test results confirmed that the compared groups did not differ from each other.

### 3.3. Age and the Concentration of Ethyl Alcohol

The calculated r Pearson correlation analysis also showed that the age of victims correlated with the alcohol concentration; r = 0.160, *p* < 0.01. There was a linear relationship between the age of the victims and the alcohol concentration in the body. This relationship was of a relatively low strength. The older the victims, the higher the alcohol concentration. The same analysis performed for each season showed that a statistically significant correlation between the age of victims and alcohol concentration occurred only in the spring months (March, April and May). In other periods (winter, summer and autumn), the statistical analysis did not prove a significant association between the age of suicide victims and the alcohol concentration (Table 2).

It was also found that there was no statistically significant correlation between the season of the year and the alcohol concentration in suicide victims; *p* = 0.274. Both in the case of younger victims and victims over 40 (the criterion for dividing into two groups was the median age Me = 40), there were no significant differences between the average levels of alcohol concentration (*p* = 0.686 vs. *p* = 0.354) (Table 3).

### 3.4. Gender and Season

The analysis conducted using the chi square independence test showed a relationship between gender and season of the year—χ^2^(3) = 8.64; *p* < 0.05; *V*_cramer’s_ = 0.166. However, this is a relatively weak relationship. The proportions summarised in Table 4 show more frequent suicide in women in September, October and November compared to other months (42.9% among women). In the group of men, most often, suicides occurred in the spring months (March, April and May), and they constituted 30.8% in this group. It is worth noting that the strongest period in the group of men (spring months) is the period with the lowest percentage of suicides in the group of women (only 10.7%) (Table 4).

### 3.5. Age and Season

Considering the age of victims broken down in two age ranges, i.e., ≤40 years and >40 years, and the division of months into four groups corresponding with four seasons of the year, i.e., winter (December, January and February), spring (March, April and May), summer (June, July and August) and autumn (September, October and November), the examination of a relation between age of victims and the season did not prove the existence of a statistically relevant influence. However, a certain tendency was observed. Over 56% of all younger victims (≤40 years) died in spring or summer. The distribution in older victims (>40 years) was more balanced (Table 5).

## 4. Discussion

There are few publications on the relationship between seasonal peaks of suicides (a peak meaning a staggering rise in suicide rates) and the occurrence or exacerbation of mental disorders (e.g., mood disorders, disorders related to the abuse of psychoactive substances, including ethyl alcohol, or disorders associated with schizophrenia). In a study conducted in Sweden, seasonal growth in suicide cases in spring and early summer was observed in patients with diagnosed neuroses [11]. Rocchi et al. also described the seasonality of suicides among patients with mental illness, while Postolache et al. reported an increase in the number of suicides in spring among victims with mood disorders [12,13]. Another study conducted by Kim et el. showed a spring–summer peak among patients with depression and an autumn–winter peak among patients with schizophrenia [14].

There is a great number of studies which indicate the occurrence of the so-called seasonality of behaviours of suicide (“temperature in spring”, Morselle) [15]. A review of studies published in the 20th century shows that the peak of suicides can be observed in the spring months, although some analyses indicate that the peak can also be observed in autumn [16]. Spring is the season with the highest number of suicides in the northern and southern hemisphere. In the United States of America and Canada, China, Pakistan, Australia, South Africa and Europe, the peak time of suicides is in spring. Similar results were also obtained in other countries [17,18,19,20,21,22,23,24,25]. However, recent research is not so clear. Although high suicide rates have been reported in Finland, Ireland and Italy in the summer, this model is much less expressed or even absent in studies conducted in England and Wales, Australia and New Zealand, Singapore and Switzerland [26,27,28,29,30,31,32].

The seasonality phenomenon also applies to suicide attempts [33]. Seasonality and temporal variations in the frequency of suicide attempts depend primarily on the sex of the victims. The results of the WHO/EURO Multicenter Study on Parasuicides indicated a seasonal peak of suicide attempts in women in spring and winter (December), but showed no significant seasonal variation in suicide attempts in men [16].

The results obtained in the presented study were compared with seasonal patterns observed in other studies, most of which confirm the occurrence of the “spring peak”, mainly for men, the elderly and violent suicide methods (which means the highest number of suicides per year), and the secondary autumn peak of suicides. The results of the presented study proved to be consistent with this pattern. The occurrence of either a spring or autumn peak was dependent on the sex of the victim.

Studies by other authors have indicated the relationship between age and seasonal variation of suicides. According to Maesa et al. the suicide rate is higher among younger people in spring (March and April), and among older people at the end of summer (August) [34]. McCleary et al. observed the peak of suicides in younger people in winter and autumn, and in older people in summer. The analysis conducted in the presented study showed no statistically significant relationship between the age of the victims and the season of the year. However, a certain tendency was observed: over half of all younger victims of suicide (≤40 years) died in spring or summer [35].

Suicide patterns vary depending on the sex of the victims [33]. In studies by other authors, only one spring peak of suicides was found for men and two peaks, i.e., one in spring and one in autumn, were observed among women [36,37,38,39,40,41]. In England, for example, “middle-aged women with school children often suicide in autumn, at the beginning of the school year…”—which means a shortened time of direct contact with a child [36,37]. The impact of sex on the seasonality of suicides was also reported in Hungary, where the spring peak was a consequence of depression-related suicides, especially among men = increased use of prescribed drugs in the population [42].

Research by the Central Statistical Office conducted in the years 1999–2003 confirmed the occurrence of the seasonality of suicides in Poland. It revealed stable seasonality of suicides in three groups: general Polish population, age group 40–44 regardless of sex, and men aged 40–44. In addition, stable seasonality with a peak in spring and summer, and a clear decrease in winter was observed. The study did not show stable seasonality of suicides in women [43]. The results are comparable with the results of most studies carried out in other countries [44]. In the presented study, the spring peak of suicides in men was recorded in May, and the autumn peak of suicides among women in September.

Among many factors impacting upon the risk of suicide, it is important to define modifiable factors, one of them being alcohol dependence. According to the Polish Police Headquarters, victims under the influence of alcohol constitute the largest percentage of suicide victims. This percentage has been growing since 2009, compared to previous years [2]. In a study conducted in Sweden, Brådvik et al. revealed a seasonal spring peak of suicides in male patients with alcohol dependence [45]. The results of the analysis performed in the presented study demonstrated that the relation between the season of the year and the alcohol concentration in suicide victims was not statistically significant. The average alcohol concentration reported in men and women was comparable. It was found, however, that alcohol concentration correlated with the age of the victims, i.e., the older the victims, the higher the alcohol concentration. It is significant that this relationship was observed only in the spring months (March, April and May). This correlation suggests a causal relationship between alcohol abuse and suicide.

The results of the analysis seem to be consistent with seasonal patterns observed in other studies, and they confirm the occurrence of suicide seasonality. However, certain limitations hinder reaching unambiguous conclusions. They result from methodological and environmental differences between the studies. In order to improve the strategies of suicide prevention, it is necessary to identify factors which are related to the seasonal variation of suicidal behaviours, and to gain knowledge concerning the mechanisms behind this phenomenon.

## 5. Conclusions

- The seasonality of suicides is a phenomenon which can also be observed among suicide victims under the influence of alcohol.
- The seasonal variation of suicides is dependent on the sex of the victims.
- It was observed that the relationship between age and alcohol concentration in suicide victims can be the cause of suicide.

## Figures and Tables

**Table 1:** Distribution of analysed variables.

**Year**	**N**	**%**
2009	75	23.7
2010	63	19.9
2011	61	19.2
2012	57	18.0
2013	61	19.2
Date of post-mortem examination	*N*	%
January	20	6.3
February	18	5.7
March	28	8.8
April	27	8.5
May	37	11.7
June	33	10.4
July	29	9.1
August	21	6.6
September	28	8.8
October	23	7.3
November	25	7.9
December	28	8.8
Gender	*N*	%
Women	28	8.8
Men	289	91.2
Initial cause of death	*N*	%
Hanging	305	96.2
Suspicion of hanging	11	3.5
Other	1	0.3
Results of post-mortem examination	*N*	%
Ligature mark	311	98.1
Ligature mark and other injury	2	0.6
Other	4	1.3

**Table 2:** Relationship between level of ethyl alcohol and age of victims depending on the season.

		Age
Winter	Pearson’s r	0.074
	Significance	0.554
Spring	Pearson’s r	0.283
	Significance	0.006
Summer	Pearson’s r	0.112
	Significance	0.315
Autumn	Pearson’s r	0.156
	Significance	0.178

**Table 3:** The average alcohol concentration in suicide victims depending on the season.

	Age					
	≤40 Year			>40 Year		
	N	M	SD	N	M	SD
Winter	33	1.67	1.07	33	1.88	0.90
Spring	46	1.57	0.98	46	1.90	0.88
Summer	45	1.64	0.83	38	1.94	0.80
Autumn	37	1.42	1.02	39	1.63	0.80
Total	161	1.58	0.97	156	1.84	0.85

**Table 4:** Correlation between gender and a period in which an autopsy was carried out.

			Gender		General
			Women	Male	
Season of the year	Winter	Number	7a	59a	66
		% of gender	25.0%	20.4%	20.8%
	Spring	Number	3a	89b	92
		% of gender	10.7%	30.8%	29.0%
	Summer	Number	6a	77a	83
		% of gender	21.4%	26.6%	26.2%
	Autumn	Number	12a	64b	76
		% of gender	42.9%	22.1%	24.0%
General		Number	28	289	317
		% of gender	100.0%	100.0%	100.0%

Note: the proportions between the columns that do not share the letter index differ significantly between each other at the level of *p* < 0.05.

**Table 5:** Number and percentage of suicide victims, broken down by age, depending on the season.

			Age		Total
			≤40 Year	>40 Year	
Season	Winter	Number	33	33	66
		% of the age	20.5%	21.2%	20.8%
	Spring	Number	46	46	92
		% of the age	28.6%	29.5%	29.0%
	Summer	Number	45	38	83
		% of the age	28.0%	24.4%	26.2%
	Autumn	Number	37	39	76
		% of the age	23.0%	25.0%	24.0%
Total		Number	161	156	317 100.0%
		% of the age	100.0%	100.0%	

## References

[1] Preventing Suicide: A Global Imperative. http://www.who.int/mental_health/suicide-prevention/world_report_2014/en/.

[2] Suicides in Poland. http://statystyka.policja.pl/st/wybrane-statystyki/zamachy-samobojcze.

[3] Khemiri L., Jokinen J., Runeson B., Jayaram-Lindström N. (2016). Suicide risk associated with experience of violence and impulsivity in alcohol dependent patients. Sci. Rep..

[4] Lester D., Krysinska K.E. (2004). The regional variation of murder and suicide in Poland. OMEGA-J. Death Dying.

[5] Woo J.-M., Okusaga O., Postolache T.T. (2012). Seasonality of Suicidal Behavior. Int. J. Environ. Res. Public Health.

[6] Alsalman R., Alansari B. (2016). Relationship of suicide ideation with depression and hopelessness. Eur. Psychiatry.

[7] Coppola I., Marangon D., Gramaglia C., Delicato C., Di Marco S., Gattoni E., Venesia A., Avanzi G.C., Castello L., Bert F. (2016). In a period of economical crisis who is at risk for attempted suicide?. Eur. Psychiatry.

[8] Webb R.T., Lichtenstein P., Larsson H., Geddes J.R., Fazel S. (2014). Suicide, hospital-presenting suicide attempts, and criminality in bipolar disorder: Examination of risk for multiple adverse outcomes. J. Clin. Psychiatry.

[9] Spinazzola J., Ford J.D., Zucker M., van der Kolk B.A., Silva S., Smith S.F., Blaustein M. (2017). Survey evaluates: Complex trauma exposure, outcome, and intervention among children and adolescents. Psychiatric Ann..

[10] Sher L. (2006). Alcoholism and suicidal behavior: A clinical overview. Acta Psychiatr. Scand..

[11] Reutfors J., Ösby U., Ekbom A., Nordström P., Jokinen J., Papadopoulos F.C. (2009). Seasonality of suicide in Sweden: Relationship with psychiatric disorder. J. Affect. Disord..

[12] Rocchi M.B., Sisti D., Miotto P., Preti A. (2007). Seasonality of suicide: Relationship with the reason for suicide. Neuropsychobiology.

[13] Postolache T.T., Mortensen P.B., Tonelli L.H., Jiao X., Frangakis C., Soriano J.J., Qin P. (2010). Seasonal spring peaks of suicide in victims with and without prior history of hospitalization for mood disorders. J. Affect. Disord..

[14] Kim C.D., Lesage A.D., Seguin M., Chawky N., Vanier C., Lipp O., Turecki G. (2004). Seasonal differences in psychopathology of male suicide completers. Compr. Psychiatry.

[15] Morselli E. (1881). Suicide: An Essay on Comparative Moral Statistics.

[16] Jessen G., Andersen K., Arensman E., Bille-Brahe U., Crepet P., Leo D.D., Hawton K., Haring C., Helmeland H., Michel K. (1998). Temporal fluctuations and seasonality in attempted suicide in Europe: Findings from the who/euro multicentre study on parasuicide. Arch. Suicide Res..

[17] Kevan S.M. (1980). Perspectives on season of suicide: A review. Soc. Sci. Med. Med. Geogr..

[18] Liu Y., Zhang Y., Arai A., Obayashi Y., Tamashiro H. (2015). Gender-based seasonality of suicide in Japan, 2005–2012. Asia Pac. J. Public Health.

[19] Saeed A., Bashir M.Z., Khan D., Iqbal J., Raja K.S., Rehman A. (2002). Epidemiology of suicide in Faisalabad. J. Ayub Med. Coll. Abbottabad.

[20] De Leo D., Sveticic J., Milner A. (2011). Suicide in Indigenous people in Queensland, Australia: Trends and methods, 1994–2007. Aust. N. Z. J. Psychiatry.

[21] Bantjes J., Kagee A. (2013). Epidemiology of suicide in South Africa: Setting an agenda for future research. S. Afr. J. Psychology.

[22] Hiltunen L., Suominen K., Lönnqvist J., Partonen T. (2011). Relationship between day length and suicide in Finland. J. Circadian Rhythms.

[23] Sisti D., Rocchi M.B., Macciò A., Preti A. (2012). The epidemiology of homicide in Italy by season, day of the week and time of day. Med. Sci. Law.

[24] Wong Y.J., Maffini C.S., Shin M. (2014). The racial-cultural framework: A framework for addressing suicide-related outcomes in communities of color. Couns. Psychol..

[25] Christodoulou C., Efstathiou V., Bouras G., Korkoliakou P., Lykouras L. (2012). Seasonal variation of suicide. A brief review. Encephalos.

[26] Coimbra D.G., e Silva A.C.P., de Sousa-Rodrigues C.F., Barbosa F.T., de Siqueira Figueredo D., Santos J.L.A., Barbosa M.R., de Medeiros Alves V., Nardi A.E., de Andrade T.G. (2016). Do suicide attempts occur more frequently in the spring too? A systematic review and rhythmic analysis. J. Affect. Disord..

[27] Ramamurthy P., Thilakan P. (2019). Geographical and temporal variation of suicide in India, 2006–2015: An investigation of factors associated with suicide risk difference across states/union territories. Indian J. Psychol. Med..

[28] Rocchi M.B., Sisti D., Cascio M.T., Preti A. (2007). Seasonality and suicide in Italy: Amplitude is positively related to rates. J. Affect. Disord..

[29] Yip P., Chao A., Chiu C. (2000). Seasonal variation in suicides: Diminished or vanished. Experience from England and Wales, 1982–1996. Br. J. Psychiatry.

[30] Yip P., Chao A., Ho T.P. (1998). A re-examination of seasonal variation in suicides in Australia and New Zealand. J. Affect. Disord..

[31] Parker G., Gao F., Machin D. (2001). Seasonality of suicide in Singapore; data from the equator. Psychol. Med..

[32] Yang A.C., Tsai S.J., Huang N.E. (2011). Decomposing the association of completed suicide with air pollution, weather, and unemployment data at different time scales. J. Affect. Disord..

[33] Mergl R., Havers I., Althaus D., Rihmer Z., Schmidtke A., Lehfeld H., Niklewski G., Hegerl U. (2010). Seasonality of suicide attempts: Association with gender. Eur. Arch. Psychiatry Clin. Neurosci..

[34] Maes M., Cosyns P., Meltzer H.Y., De Meyer F., Peeters D. (1993). Seasonality in violent suicide but not in non violent suicide or homicide. Am. J. Psychiatry.

[35] McCleary R., Chew K.S., Hellsten J.J., Flynn-Bransford M. (1991). Age-and sex-specific cycles in United States suicides. Am. J. Public Health.

[36] Moore F.R., Bell M., Macleod M., Smith E., Beaumont J., Graham L., Harley T.A. (2018). Season, weather, and suicide–Further evidence for ecological complexity. Neurol. Psychiatry Brain Res..

[37] Brewerton T.D., Putnam K.T., Lewine R.R., Risch S.C. (2018). Seasonality of cerebrospinal fluid monoamine metabolite concentrations and their associations with meteorological variables in humans. J. Psychiatr. Res..

[38] Amritwar A.U., Lowry C.A., Brenner L.A., Hoisington A.J., Stiller J.W., Hamilton R., Postolache T.T. (2017). Mental health in allergic rhinitis: Depression and suicidal behavior. Curr. Treat. Opt. Allergy.

[39] Hawton K., Saunders K.E., O’Connor R.C. (2012). Self-harm and suicide in adolescents. Lancet.

[40] Preti A., Miotto P., Coppi M.D. (2000). Season and suicide: Recent findings from Italy. Crisis.

[41] Shinsugi C., Stickley A., Konishi S., Ng C.F.S., Watanabe C. (2015). Seasonality of child and adolescent injury mortality in Japan, 2000–2010. Environ. Health Prev. Med..

[42] Sebestyen B., Rihmer Z., Balint L., Szokontor N., Gonda X., Gyarmati B., Bodecs T., Sandor J. (2010). Gender differences in antidepressant use-related seasonality change in suicide mortality in Hungary, 1998–2006. World J. Biol. Psychiatry.

[43] Młodozeniec A., Brodniak W.A., Polewka A., Bembenek A. (2010). Seasonality of suicide in Poland. Analysis of the Main Statistical Office data for the years 1999–2003. Psychiatr. Pol..

[44] Van Orden K.A., Witte T.K., Cukrowicz K.C., Braithwaite S.R., Selby E.A., Joiner T.E. (2010). The interpersonal theory of suicide. Psychol. Rev..

[45] Brådvik L., Berglund M. (2002). Seasonal distribution of suicide in alcoholism. Acta Psychiatr. Scand..

